# The Impact of Digital Devices on Children’s Health: A Systematic Literature Review

**DOI:** 10.3390/jfmk9040236

**Published:** 2024-11-14

**Authors:** Valentina Presta, Alessandro Guarnieri, Fabiana Laurenti, Salvatore Mazzei, Maria Luisa Arcari, Prisco Mirandola, Marco Vitale, Michael Yong Hwa Chia, Giancarlo Condello, Giuliana Gobbi

**Affiliations:** 1 Department of Medicine and Surgery, University of Parma, Via Gramsci 14, 43126 Parma, Italy; valentina.presta@unipr.it (V.P.); alessandro.guarnieri@unipr.it (A.G.); or fabiana.laurenti@univr.it (F.L.); salvatore.mazzei@unipr.it (S.M.); marialuisa.arcari@unipr.it (M.L.A.); prisco.mirandola@unipr.it (P.M.); giuliana.gobbi@unipr.it (G.G.); 2 Department of Neurosciences, Biomedicine and Movement Sciences, University of Verona, Piazzale Ludovico Antonio Scuro 10, 37124 Verona, Italy; 3 Faculty of Medicine and Surgery, University Vita-Salute San Raffaele, Via Olgettina 58, 20132 Milan, Italy; vitale.marco@hsr.it; 4 Physical Education and Sports Science Academic Department, National Institute of Education, Nanyang Technological University, 1 Nanyang Walk, Singapore 637616, Singapore; michael.chia@nie.edu.sg

**Keywords:** screen time, physical activity, body composition, sleep disturbances, coronavirus

## Abstract

Background: The impact of prolonged digital device exposure on physical and mental health in children has been widely investigated by the scientific community. Additionally, the lockdown periods due to the COVID-19 pandemic further exposed children to screen time for e-learning activities. The aim of this systematic review (PROSPERO Registration: CRD42022315596) was to evaluate the effect of digital device exposure on children’s health. The impact of the COVID-19 pandemic was additionally explored to verify the further exposure of children due to the e-learning environment. Methods: Available online databases (PubMed, Google Scholar, Semantic Scholar, BASE, Scopus, Web of Science, and SPORTDiscus) were searched for study selection. The PICO model was followed by including a target population of children aged 2 to 12 years, exposed or not to any type of digital devices, while evaluating changes in both physical and mental health outcomes. The quality assessment was conducted by using the Joanna Briggs Institute (JBI) Critical Appraisal Tool. Synthesis without meta-analysis (SWiM) guidelines were followed to provide data synthesis. Results: Forty studies with a total sample of 75,540 children were included in this systematic review. The study design was mainly cross-sectional (n = 28) and of moderate quality (n = 33). Overall, the quality score was reduced due to recall, selection, and detection biases; blinding procedures influenced the quality score of controlled trials, and outcome validity reduced the quality score of cohort studies. Digital device exposure affected physical activity engagement and adiposity parameters; sleep and behavioral problems emerged in children overexposed to digital devices. Ocular conditions were also reported and associated with higher screen exposure. Home confinement during COVID-19 further increased digital device exposure with additional negative effects. Conclusions: The prolonged use of digital devices has a significant negative impact on children aged 2 to 12, leading to decreased physical activity, sleep disturbances, behavioral issues, lower academic performance, socioemotional challenges, and eye strain, particularly following extended online learning during lockdowns.

## 1. Introduction

Technology is becoming easily accessible and required for many aspects of everyday life, independently of age. Technological devices, such as mobile phones, tablets, and laptops, are commonly used for communication, dissemination, and education, but abuse and/or addictive behavior is becoming predominant [1]. The more children grow, the more they consider themselves “experts” in the field of technology, claiming an extensive use of technological devices among their peers [2]. Children born into a digitalized world have been defined as “digital natives”; however, this does not guarantee that they will automatically develop digital skills, nor that their behavior toward digital devices will not be negatively influenced [3]. It is unclear if the (ab)use of such devices can actually be considered as part of an “addictive” behavior [4]: technology allows to log in social networks for chatting or gaming activities, videos watching, and music listening, but nowadays, working and educational aspects should also be considered. Regardless of reasons, studies show an increased use of digital devices and media in the last decades, influencing the emotional and behavioral sphere of individuals [5]. Therefore, the emerging literature reported a variety of health repercussions in both preschoolers (2–5 years old) and school-aged children (6–12 years old), including body image and sleep issues, anxiety, depression, and poor academic performances [4].

Children aged 2 to 12 years are particularly exposed to digital devices [6], which may worsen the already increasing trend of sedentary behavior during childhood [7,8]. Digital devices impact children’s daily lives by forcing prolonged inactivity [8], and internet addiction has been linked to anxiety, depression, and eating disorders in middle and late childhood [7,9]. Similar effects have been linked to the “hikikomori” condition, characterized by severe social isolation during childhood. Particularly in this context, the anxiety and depression symptoms are worsened by virtual interactions and digital communication, which, in turn, increase social withdrawal [10]. Prolonged sitting time, or time spent in “non-exercise” activities, is associated with a higher risk of cardiovascular and metabolic diseases [11]. In contrast, physical activity helps reduce overweight/obesity and related health risks [12,13,14]. The recent WHO’s guidelines emphasize the need to reduce screen time, which is connected to increased sedentary behavior [15]. Additionally, childhood lifestyle choices affect health in adulthood [12,16] and are influenced by various factors [17]. In particular, an umbrella systematic literature review on behavioral determinants demonstrated that “screen use” was negatively associated with moderate-to-vigorous physical activity, whilst “previous physical activity” and “independent mobility and active transport” were positively associated with overall physical activity in children [18]. As a matter, WHO and the American Academy of Pediatrics agree with a total screen time of 1 h/day in children aged 2–5 years. A certain tolerance is given from 6 years old to adolescence due to educational use of digital devices, whereas avoidance or limit use/hours spent in digital devices is strongly recommended under 2 years of age [19]. However, evidence shows that children in several parts of the world do not meet neither the recommended amount of physical activity nor the screen time guidelines [14,20,21,22].

A healthy lifestyle in children depends on exercise engagement, a decrease in sitting time, sleep quality, and nutrition habits [23,24]. Similarly, sleep duration is also associated with better body composition, academic performance [25], and emotional well-being [26]. Interestingly, a longer screen time shortened the sleep duration, negatively influencing the cognitive [27], socio-relational, and physical growth during childhood [24]. Recently, Lissak [28] highlighted the adverse effects of screen time exposure during childhood. Among these, accumulating prolonged sitting time increases cardiovascular risks (causing hypertension, an unfavorable lipidic profile, and overweight/obesity). These results are reported for a screen time amount (leisure activities such as TV watching or gaming) of 2 to 3 h/day. Vision discomfort and musculoskeletal symptoms were also reported, as well as neurological and behavioral disorders [28,29,30].

A general increase in digital device use was reported due to the massive lockdown and quarantine measures during the COVID-19 pandemic [31]. Smart working strategies were applied worldwide to ensure work activities even during home confinement [32]. Children were involved in e-learning methodologies, further increasing the digital media exposure and screen time. Wang et al. [33] reported that children were negatively affected by the lockdown due to the lack of outdoor activities and peer interactions. In addition, the increased screen time led to a routine disruption with sleep disorders and daytime stress [34]. However, children can also benefit from digital environments due to the quicker access to current news, family engagement in digital game sharing, peer connections, and learning components. Recently, the WHO released a position statement on how children can grow safely in an ever-expanding digital environment. In this statement, the WHO also emphasizes the positive aspects of technology, such as easy access to information, social connections, the promotion of digital literacy, and self-expression supporting creativity. However, depending on the amount and nature of digital exposure, these positives can become self-defeating, increasing health risks for children and impacting various aspects of their development [35]. Therefore, the realistic needs of digital devices for educational and social purposes and the balance between positive and negative consequences should be carefully debated.

Given this background, this study has two objectives: (i) the first aims to systematically review the available evidence about the impact of digital device exposure on children’s physical and mental health; (ii) and the second is to investigate the effects of the lockdown period and e-learning methodologies on screen time and health indicators in children. To the best of our knowledge, no systematic reviews have been previously conducted to verify the effects of exposure to digital devices on children’s health. Finally, the evidence could support an in-depth study on how digital equipment should be managed to minimize its influence on physical and mental health during childhood.

## 2. Materials and Methods

### 2.1. Protocol Registration

This systematic review was registered 5 in the International Prospective Register of Systematic Reviews (PROSPERO; CRD4202231596; July 2022).

### 2.2. Search Strategy

The literature search was performed from July 2022 to April 2023 across the following databases: PubMed, Google Scholar, Semantic Scholar, BASE, Scopus, Web of Science, and SPORTDiscus. Screening was performed according to the Preferred Reporting Items for Systematic Reviews and Meta-Analyses (PRISMA) [36]. The following keywords and Boolean operators were used as search terms: (motor development OR motor coordination) AND (digital media OR screen time) AND children; (e-learning OR sleep disorder) AND (digital device OR digital media) AND children; (physical OR mental) AND (cognitive OR mental) AND (health OR development) AND child AND digital media. The identified articles were screened and assessed for eligibility criteria. The references to the selected articles were also considered. Following the PICO model (Population, Intervention, Comparator, Outcomes), the target population was children aged 2 to 12 years. The intervention, defined as exposure to digital devices, included tablets, smartphones, TVs, laptops, and game consoles. The comparator group consisted of children who were not exposed to any digital devices. The main outcomes included changes in both mental and physical health due to exposure to digital devices.

### 2.3. Eligibility Criteria

Studies assessing the impact of digital device exposure on the mental and physical health of children were considered eligible. Mental outcomes were identified as changes in cognitive function, behavior, and psychosocial responses of children exposed to a digital environment. Physical outcomes about physical activity levels, motor skills, posture, and body composition were included. Sleep habits and COVID-19-related change in both mental and physical outcomes were additionally investigated. No restrictions regarding publication dates or types of digital devices were applied. Studies were also screened for meeting the following inclusion criteria: (i) studies involving children aged 2–12 years old; (ii) absence of physical or mental illness; (iii) available full text in English.

### 2.4. Data Extraction

The data extraction process was conducted independently by two authors (V.P. and A.G.), and disagreements were verified by a third one (GC). A summary table is provided as supplementary material with the aim, design, and main findings of the included studies (Supplementary Table S1).

### 2.5. Quality Assessment

The Joanna Briggs Institute (JBI) Critical Appraisal Tool was used to assess the quality and risk of bias of the included studies [37]. The JBI Critical Appraisal Tool included 8 to 11 items that varied according to the study design (i.e., randomized controlled trials (RCTs), cross-sectional studies, and cohort studies). Thus, by using the same tool, it was possible to assess the quality of the studies uniformly and independently of the design. Each item consisted of “yes”, “no/unclear”, “not applicable”, or “not reported” answers. A score was provided for only positive or negative/unclear answers: 1 point for “yes”, 0 points for “no/unclear”. The total score of each study was converted in percentages, and the quality score was evaluated as follows: a score > 80% for high-quality studies; 50–79% for moderate quality score; <50% low-quality studies.

### 2.6. Data Synthesis

Quantitative analysis (meta-analysis) was precluded due to the heterogeneity of the study characteristics, methodological approaches, and number of studies for outcomes measured. Therefore, the data synthesis was performed according to the Synthesis-Without Meta-Analysis (SWiM) reporting guidelines [38]. In the current review, outcomes associated with digital device exposure were synthesized in 5 areas: (1) physical activity and body composition; (2) motor skills and posture; (3) sleep habits; (4) behavioral aspects; and (5) COVID-19 pandemic effect with visual function assessment. Considering the variety of terminology commonly used in this area of research, in the current review, the following terms were used: digital devices (alternatively screen), referring to the unique tool as TV, smartphones, laptops, and tablets; digital media, referring to any content that relies on an electronic device for its creation, distribution, viewing, and storage, such as software, video games, videos, websites, social media, and online advertising; screen time, referring to the measured outcome for digital device or digital media exposure and expressed in hours per day spent on digital devices on a weekday and/or weekend.

## 3. Results

### 3.1. Study Selection

**Figure 1:**
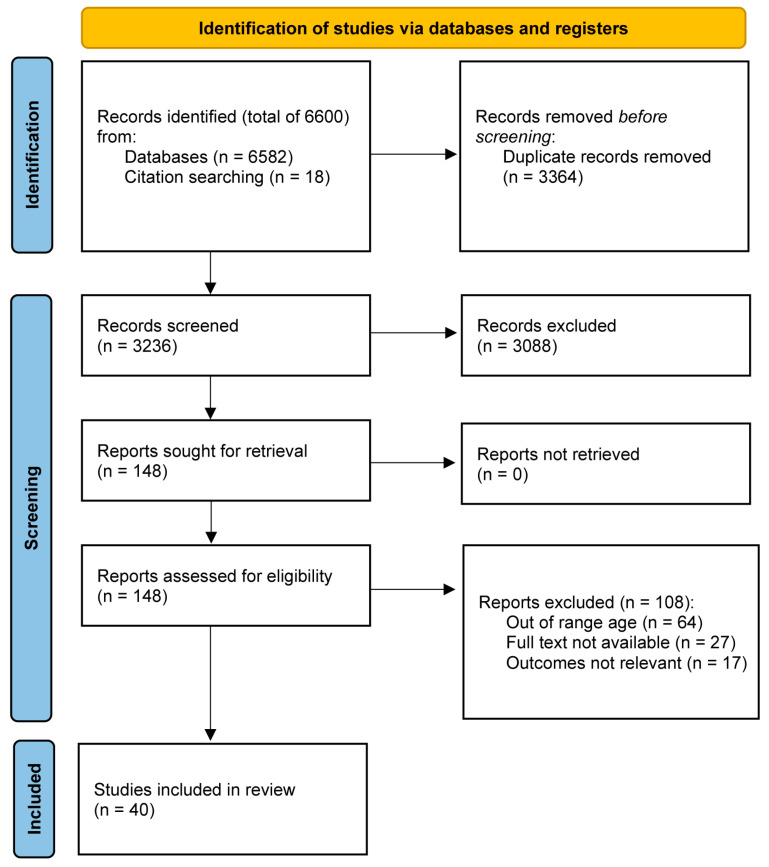
Preferred Reporting Items for Systematic Reviews and Meta-Analyses (PRISMA) flow diagram from the identification of the inclusion of studies in the systematic review.

A total of 6600 studies were identified, and 3364 duplicates were removed. The remaining 3236 were screened for title and abstract, among which 148 were eligible. After considering the eligibility criteria, 108 articles were excluded for multiple reasons, and a final number of 40 studies were included in the systematic review (Figure 1).

### 3.2. Study Characteristics

The study characteristics are reported in a table as supplementary material (Supplementary Table S1). The publication dates of the studies ranged between 2012 and 2022. Most of the included studies (n = 28) adopted a cross-sectional design [20,39,40,41,42,43,44,45,46,47,48,49,50,51,52,53,54,55,56,57,58,59,60,61,62,63,64,65], whilst the remaining were randomized controlled trials (RCTs, n = 3) [66,67,68] and cohort studies (n = 9) [30,69,70,71,72,73,74,75,76]. Many of the included cross-sectional studies adopted surveys and questionnaires to investigate screen time exposure and/or the related effect on the identified outcomes.

Australia [47,48,54,61,68,75] and China [30,61,65,69,70,71] had the most studies (n = 6 studies for each country), followed by Canada (n = 5) [20,45,49,72,74], Germany (n = 4) [62,66,67,73], Japan (n = 3) [53,58,60], Italy (n = 3) [40,61,62], Finland (n = 2) [51,52], Singapore (n = 2) [46,64], Spain (n = 2) [50,62], United Kingdom (n = 2) [61,63], and USA (n = 2) [57,61]. Belgium, Cyprus, England, Estonia, Hungary, Ireland, Korea, Portugal, Russia, Sweden, Switzerland, Tunisia, and Turkey were also represented with a single study [39,41,42,43,44,55,56,57,61,62,76]. Two cross-sectional studies involved simultaneously six [61] and eight [62] countries. One cross-sectional study did not report the sample country [59].

Age ranged from 2 to 12 years old across the studies. Studies including preschool children (from 2 to 5 or 6 years old—children in Germany can attend preschool until they are 6 years old) were the majority (n = 17) [20,40,42,44,46,47,51,52,53,54,65,66,67,72,74,75], followed by studies on school-aged children (6–12 years old) (n = 14) [30,41,43,45,49,50,57,58,60,63,68,69,70,71]. The remaining studies included a sample of both preschoolers and school-aged children (n = 9) [39,48,55,56,59,61,62,64,76].

The sample size of the included studies ranged from 10 to 15,330. A total of 7 studies included a sample ≤ 100 children [39,40,48,54,60,66,68]; 18 studies included a sample from 101 to ≤1000 children [20,30,42,43,44,45,47,50,52,56,57,59,67,70,71,73,75,76]; 12 studies included a sample from 1001 to ≤10,000 children [49,51,53,55,58,61,63,64,65,69,72,74]; 2 studies included over 10,000 children (13,397 and 15,330, respectively) [41,62], whereas 1 study did not report the sample size [46].

For gender, all of the included studies evaluated both females and males, but only three studies investigated gender differences [39,41,43]. Two studies did not report the gender of the sample [46,65].

Parental socio-demographics were investigated in over half of studies: at least one among educational level, employment and socioeconomic status, language spoken, nationality, and ethnicity was included [20,39,40,41,44,45,46,47,48,49,51,52,53,56,59,61,62,63,72,74,75,76]. Studies recruiting only mothers reported also maternal age and marital status [42,47,72,74]. Area of residence was specified in only four studies [41,48,49,51]. The remaining 17 studies did not specify the sample demographics [30,43,50,54,55,57,58,60,64,65,66,67,68,69,70,71,73].

#### 3.2.1. Intervention

Although the majority of the included studies consisted of a cross-sectional design, only four studies [54,66,67,68] were conducted with an experimental design. In particular, two studies explored writing performance by comparing the effect of a handwriting and/or typewriting training program in 4-to-6-year-old children [66,67]. One study evaluated sedentariness during an 8-week intervention with the removal or replacement of electronic games with active input games [68]. A cross-sectional laboratory trial analyzed children’s body postures while playing with digital or material toys and during TV watching [54].

#### 3.2.2. Measurement of Digital Device Exposure

All of the included studies evaluated the digital device exposure throughout various procedures. Screen time (i.e., hours per day spent with digital devices on a weekday and/or weekend) was the most evaluated parameter. Some studies also investigated the weekly frequency and timing (i.e., before, after bedtime, at night, and before and after home confinement during COVID-19). Preschool samples were analyzed via parent (or caregivers/guardians/teachers) report answering about media habits, types, number, and position (i.e., bedroom) of devices (for example, via 3-day or 7-day diary); children were directly involved if they were able to understand and answer questions about their digital device exposure.

The exposure to multiple digital devices (or media multitasking, referring to two or more devices used concurrently) was investigated to explore the effects on children’s behavioral responses [43,56,57]. At the same time, content and quality of media engagement were investigated [41,42,47,61,64], reporting similar tendencies in media use for entertainment and leisure activities as compared with educational or learning apps [41,62,64].

A new questionnaire was developed and validated in 2019 by Chia and colleagues [77]. The Surveillance of screen Media hAbits in earLy chiLdhood Questionnaire (SMALLQ^®^) was used in only three out of forty studies [46,51,64]. The SMALLQ^®^ consisted of 25 items to assess the media habits of both parents and children: questions included the digital and non-digital behavior of children and parents, time spent with any of digital devices—television, mobile, computer, video games, etc.—sleep habits and eyesight of children, along with parental awareness of digital media use.

Finally, digital device exposure was indirectly explored while detecting other primary outcomes. Thus, in the cross-sectional laboratory trial [54] and RCTs included [66,67,68], body posture was measured while using digital or non-digital devices, handwriting was compared with typewriting on digital devices, and the removal or replacement of digital devices was compared to assess behavioral changes in children in the presence or absence of a digital setting. A recent study [40] evaluated verbal production (private and social speech), while children did the same task in material and digital versions.

### 3.3. Outcomes

#### 3.3.1. Physical Activity and Body Composition

As a primary outcome, physical activity (PA) and body composition were investigated in eight studies [20,46,47,57,58,62,63,68], while five studies explored the sedentariness of the included sample, along with parameters related to weight status and adiposity [20,39,49,50,51].

PA behavior was collected with parent- and/or children-reported questionnaires (SMALLQ^®^, WHO Health Behavior School-Aged Children [HBSC], Ricci and Gagnon sedentary behavior questionnaire). Chaput et al. [20,45] used an accelerometer during 7 consecutive days to objectively categorize children engaged in low, moderate, and vigorous PA. Two studies compared individual PA levels with guidelines [20,46]: (i) the Canadian Movement Guidelines suggest at least 180 min of PA, with at least 60 min of moderate-to-vigorous PA, engagement in no more than 1 h of screen time, and sleep duration between 10 and 13 h among 3–4-year-old preschoolers; (ii) the 24-WHO Guidelines recommend having at least 180 min of PA, engaging in less than 60 min of screen time, and having 10–13 h of good quality sleep among 2-to-6-aged children. Both studies reported a low percentage of children who met those guidelines [20,46]. The multicentric study of Santaliestra-Pasías et al. [62] also considered reference guidelines (the American Academy of Pediatrics recommends a screen time exposure ≤ 2 h/d), showing a reduction in sports club or outdoor PA for children exceeding the recommended screen time. Moreover, studies investigating PA levels and digital device use reported a reduction in PA engagement because of higher screen time duration. Indeed, in a RCT, Straker et al. [68] investigated PA behavior of children who did not have access to digital devices, children who had access to electronic devices with active input games, and a control group who had access to digital devices) for 8 weeks. The results favored higher levels of outdoor PA in the absence of digital devices or with active objectives on digital device use.

Body composition was assessed to verify the associations among digital devices and body mass indicators. Body mass index (BMI) was mainly considered to evaluate the relationship with digital device exposure (n = 4) [45,47,50,57,58]. Some studies identified the BMI *z*-score [20,45,49,63] adjusted for children’s age and gender [78]. The diversity in body composition measurements included also the calculation of BMI from self- or parent-reported anthropometrics (height and body mass) [47,58] and direct and single measurements of children’s height and body mass [20,49,57], while three studies opted for the bioimpedance analysis [45,50,63]. The results across studies reported higher values for BMI and BMI *z*-score if digital device exposure increased in media multitasking activities [57] or if children had access to more devices in personal spaces (bedroom) [45,49]. Among the studies evaluating PA levels and BMI, two of them also explored food habits. Cox et al. [47] asked parents about the food consumed while children were watching TV—mainly food poor of nutrients or takeaway food—reporting an increased energy intake with consequent risk of overweight and obesity. González-Valero et al. [50] measured the adherence to the Mediterranean diet (KIDMED) in their sample, reporting poor compliance and association with increased BMI.

#### 3.3.2. Motor Skills and Posture

Motor skills and posture were investigated in four studies to examine a possible effect of digital device exposure [48,54,60,73]. In these studies, parents (tutor or caregivers) and/or children reported information about their media usage (daily screen time on a typical weekday and during the weekend and types of devices). Fine motor skills (e.g., manual dexterity) were assessed with Movement Assessment Battery for Children (M-ABC) in two studies [60,73] (second edition of M-ABC-2 in Nobusako et al. [60]); Nobusako et al. [60] additionally tested children with a visual–tactile temporal order judgment task to assess perceptual biases (children were asked to determine which stimulus—visual or tactile—was presented as the first one). Dadson et al. [48] conducted the motor assessment with a test battery and questionnaires on fine motor skills, in-hand manipulation, visual-motor integration, sensory processing, and parent-reported play skills (Bruininks–Oseretsky Test of Motor Proficiency, 2nd Edition; Test of In-Hand Manipulation—Revised; Beery Buktenica Developmental Test of Visual-Motor Integration, 6th Edition; Sensory Processing Measure—Home Form; Pretend Play Enjoyment Developmental Checklist). Findings reported significant negative associations between digital device exposure and motor skills, especially screen time, which had a negative effect on children’s sensory processing [48] and manual dexterity [60]. Developmental characteristics of motor skills are reduced across time in children usually exposed to digital devices [73]. A cross-sectional laboratory trial was designed to assess body postures while children played with digital tablets, toys, or during passive TV watching.

Digital playing elicited greater variations in arm, head, and trunk joint angles compared to TV watching but not compared with non-digital or toy-playing conditions [54].

#### 3.3.3. Sleep Habits

Nine studies investigated sleep habits and quality to ascertain the association between sleep disorders and digital device exposure [20,39,45,46,49,50,52,63,65]. A single study objectively evaluated the sleep habits of children with a 7-day worn accelerometer [45], and three studies used questionnaires, as the Children’s Sleep Habits Questionnaire (CSHQ) [52,65] and the Pittsburgh Sleep Quality Index (PSQI) [39], whereas the other studies evaluated sleep using a parent-reported survey. The presence of any device in children’s personal space (i.e., bedroom) was assessed, and it was associated with screen time in two studies [45,49], demonstrating that the higher the screen number, the lower the sleep efficiency (sleep efficiency refers to the percentage of total sleep time divided by the total time in bed) [45]. Similarly, sleep quality was reduced (namely, if children snore, wake up during the night, feel unrefreshed the day after, feel sleepy during the daytime) [49], and sleep duration was shortened with later bedtimes [49,52] in children usually exposed to digital devices during the hour before bedtime. Differently, sleep parameters remained normal in children who read a book in this hour [49]. A higher risk of sleep disorders was reported over the TV watching time threshold of 1 h/d [65].

The remaining studies registered sleep problems in their sample [39,63] due to a higher exposure to digital devices [50] and a higher percentage of children who did not meet guidelines about sleep duration (Canadian Movement Guidelines suggest sleep duration between 10 and 13 h; the 24-WHO Guidelines recommend having 10 to 13 h of good quality sleep) [20,46].

#### 3.3.4. Behavior, Socioemotional Well-Being, and Learning

Seventeen studies were grouped based on the investigated behavioral aspects, psychosocial responses, and cognitive development of children exposed to digital devices [40,41,42,43,50,51,53,55,56,59,64,66,67,72,74,75,76].

Three studies investigated behavioral aspects and control abilities [43,74,75]. The Strength and Difficulties Questionnaire (SDQ) was the prevalent tool used to measure children’s psychosocial health, covering five domains: conduct problems, hyperactivity, emotional problems, peer problems, and prosocial behavior [67]. McArthur et al. [74] also used the Behavior Assessment System for Children (BASC-II), which covers behavioral aspects as internalizing (i.e., anxiety, depression, somatization, etc.), externalizing (i.e., aggression, hyperactivity, etc.), and adaptive behavior (i.e., activities of daily living, adaptability, functional communication, and social skills). McNeill et al. [75] additionally assessed the executive functions of children using the Early Years Toolbox (device-based tasks about working memory, phonological working memory, inhibition, and shifting). The studies reported that the behavior-related parameters were associated with higher patterns of screen exposure (regardless of passive—TV watching—or active interactions, such as phones and tablets) [43,74,75]. In fact, higher levels of app use (active interaction) were associated with behavior (especially externalizing problems) and psychological difficulties, while program viewing (on TV or any other device) exerted lower inhibition (poor regulatory behavior) [75]. Similarly, higher levels of externalizing symptoms (aggression and inattention) and lower adaptation (age-appropriate life skills, leadership) were reported by McArthur et al. [74].

Self-regulation skills and self-concept were also investigated in two studies [42,50]. Self-regulation deals with behavioral control abilities (i.e., controlling emotion, thoughts, and actions in contrast to emotional explosion and aggression), by which individuals systematically control themselves [42]. Self-concept development contributes to creating a personal identity [50]; therefore, self-regulation and self-concept skills were examined by questionnaires (Self-Regulation Skills of 4–6-Year-Old Children—Mother Form and Questionnaire of Experiences Related to Video Games) along with digital device exposure. Children usually exposed to any digital devices had lower scores in the self-regulation point scale, especially among the sample preferring violent rather than educational content [42]. When evaluated, video games also affected psychosocial variables. For this reason, Gonzalez-Valero et al. [50] used the Questionnaire of Experiences Related to Video Games to evaluate expressions such as denial, concern, tolerance, and loss of control while playing video games [79]. The results showed lower scores and poor self-concept in children with a higher exposure to digital devices.

Media multitasking affected the behavioral responses of children, together with psychological distress and socioemotional functioning [43]. Emotional well-being was primarily evaluated in four studies [41,53,56,59]. SDQ was administered to parents to investigate the emotional dimension of their children [41,53,56]. Nabi & Wolfers [59] evaluated emotional intelligence variations due to digital device exposure. Emotional intelligence refers to a group of mental abilities that allow an individual to recognize and regulate emotional states and to plan, motivate, and achieve goals by using emotions [59]. Studies confirmed socioemotional dysregulation (problems in conduct and hyperactivity/inattention as measured with the SDQ) with an increase in digital device exposure [41,53,56]. Gender differences were also detected by Bohnert & Gracia [41], with the male sample demonstrating socioemotional functioning even if engaged in educational digital sessions, while the female sample was mostly affected by mobile ownership. By contrast, no relationships were reported by Nabi & Wolfers [59] between emotional intelligence parameters and digital device exposure, though higher emotional intelligence levels were found to be associated with reading activities.

Children’s behavior was also associated with parental influence and awareness regarding digital media exposure [51,55,59,64]. The digital media habits of children and parents were assessed with the SMALLQ^®^ [52,64] or by registering the screen time on a typical weekday and weekend. The results showed significant and positive associations between parents’ digital media habits and digital behavior in early childhood [51,55,59,64], since digital media co-participation reduces as children become older [51].

Lastly, cognition development, processing, and learning outcomes were assessed in eight studies [40,43,58,66,67,72,74,76]. Children’s development was assessed by (i) the Ages and Stages Questionnaire, Third Edition (ASQ-3) [72,74], consisting of five domains (i.e., communication, gross motor, fine motor, problem solving, and personal-social); (ii) the Understanding of Similar Sounding Words, evaluating the phonological memory and Raven’s matrices for non-verbal fluid intelligence [76]; (iii) verbal production assessment [40]; (iv) academic performances reported by teachers [43,58]; and (v) tests on writing performances [66,67]. Studies evaluating the directional association between screen time and academic performance reported poor levels of developmental achievement, attention, motivation, and grades with higher screen time [43,58,72,74]. Verbal production was assessed by recording private (self-talk) and social speech while children were playing the same task in a digital and material version. Passive TV watching and interactive screen devices were compared to evaluate verbal information processing. The results showed that private speech decreased during digital play sessions [40], just as passive screen exposure reduced the ability to process verbal information [76]. Digital influence was finally assessed and compared with material tasks in two RCTs evaluating writing acquisition progress after a 7-week training period [66,67]. Kiefer et al. [66] compared handwriting vs. typewriting, while Mayer et al. [67] additionally compared the third condition of handwriting with stylus on a tablet. Both studies reported better writing performances favored by handwriting training (handwriting with stylus did not significantly differ from the other conditions), with improvement in letter acquisition and visual-spatial skills inducing cognitive development [67].

#### 3.3.5. COVID-19 Effect and Visual Function

Digital device exposure during the COVID-19 pandemic was assessed with the hypothesis of a meaningful increase due to home confinement promoting distance working and education. Two studies investigated the change in digital device exposure due to the COVID-19 pandemic [39,61], whereas three studies evaluated myopia progression as a derived outcome from screen exposure during the COVID-19 pandemic [69,70,71]. A vision assessment other than COVID-19 impact was also included in two studies [30,58].

Changes in digital media use were evident comparing pre- and post-lockdown periods due to the COVID-19 pandemic: screen time increased (for an average of 1 h/day) [61] and timing of digital device use increased in the hours before bedtime and after (nocturnal screen exposure) [39], which were also associated with dry eyes sensation [58]; females were more vulnerable to a higher amount of screen time during lockdowns [39]; digital media content referred mostly to entertainment purposes rather than educational ones [61].

Ocular examinations were provided in three cohort studies, in which children exposed to COVID-19 and digital devices were evaluated for myopia incidence and progression. A higher risk to develop or worsen myopia during COVID-19 exposure was reported [69]. Moreover, there were associations with the long-time online learning and digital media reading [67] limited to tablets and phones (lesser with TV or projectors) [71]. Other than the COVID-19 period, vision function was assessed in a growing e-learning environment. Poor vision was reported as a negative long-term consequence, characterized by a low level of visual acuity, visual field, and depth perception [30].

### 3.4. Risk of Bias Assessment

**Figure 2:**
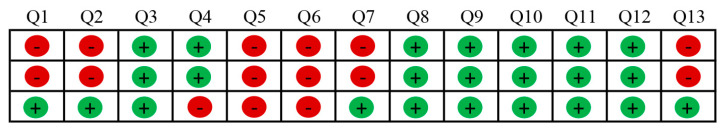
Quality assessment of randomized controlled trials (RCTs) included (n = 3, starting from the top Kiefer et al. [66], Mayer et al. [67], Straker et al. [68]), according to the Joanna Briggs Institute (JBI) Critical Appraisal Checklist for Randomized Controlled Trials [37]. 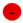 = no/unclear, 0 point; 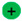 = yes, 1 point. Q1: Was true randomization used for assignment of participants to treatment groups? Q2: Was allocation to treatment groups concealed? Q3: Were treatment groups similar at the baseline? Q4: Were participants blind to treatment assignment? Q5: Were those delivering treatment blind to treatment assignment? Q6: Were outcomes assessors blind to treatment assignment? Q7: Were treatment groups treated identically other than the intervention of interest? Q8: Was follow-up completed, and if not, were differences between groups in terms of their follow-up adequately described and analyzed? Q9: Were participants analyzed in the groups to which they were randomized? Q10: Were outcomes measured in the same way for treatment groups? Q11: Were outcomes measured in a reliable way? Q12: Was appropriate statistical analysis used? Q13: Was the trial design appropriate, and any deviations from the standard RCT design (individual randomization, parallel groups) accounted for in the conduct and analysis of the trial?

**Figure 3:**
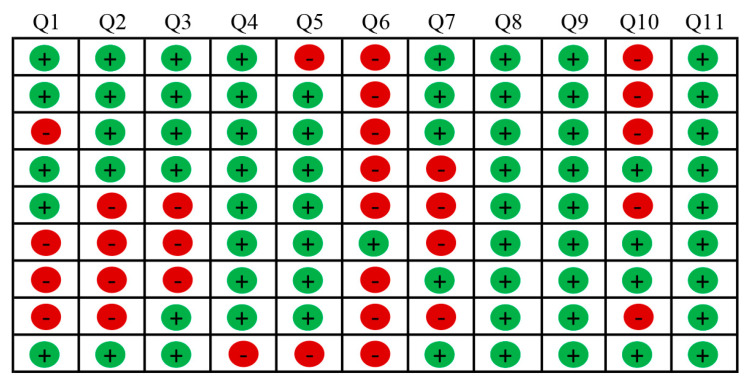
Quality assessment of cohort studies included (n = 9, starting from the top Hu et al. [69], Ma et al. [70], Ma et al. [71], Madigan et al. [72], Martzog & Suggate [73], McArthur et al. [74], McNeill et al. [75], Veraksa et al. [76], Zhang et al. [30]), according to the Joanna Briggs Institute (JBI) Critical Appraisal Checklist for Cohort Studies [37]. 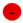 = no/unclear, 0 point; 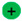 = yes, 1 point. Q1: Were the two groups similar and recruited from the same population? Q2: Were the exposures measured similarly to assign people to both exposed and unexposed groups? Q3: Was the exposure measured in a valid and reliable way? Q4: Were confounding factors identified? Q5: Were strategies to deal with confounding factors stated? Q6: Were the groups/participants free of the outcome at the start of the study (or at the moment of exposure)? Q7: Were the outcomes measured in a valid and reliable way? Q8: Was the follow-up time reported and sufficient to be long enough for outcomes to occur? Q9: Was follow-up complete, and if not, were the reasons to loss to follow-up described and explored? Q10: Were strategies to address incomplete follow-up utilized? Q11: Was appropriate statistical analysis used?

**Figure 4:**
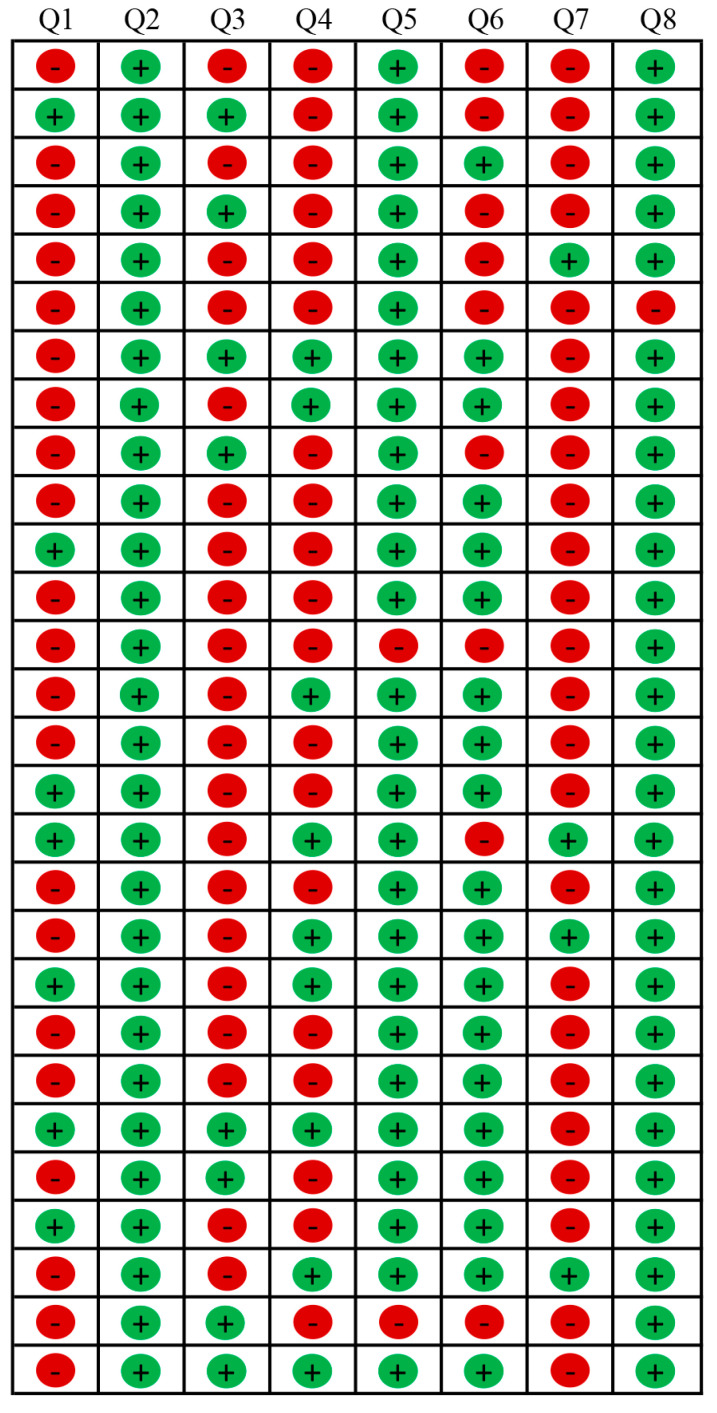
Quality assessment of cross-sectional studies included (n = 28, starting from the top Abid et al. [39], Bochicchio et al. [40], Bohnert & Gracia [41], Canaslan-Akyar & Sungur [42], Cardoso-Leite et al. [43], Chang et al. [44], Chaput et al. [45], Chaput et al. [20], Chia et al. [46], Cox et al. [47], Dadson et al. [48], Dube et al. [49], Gonzalez-Valero et al. [50], Hasanen et al. [51], Hiltunen et al. [52], Hosokawa & Katsura [53], Howie et al. [54], Jago et al. [55], Kostyrka-Allchorne et al. [56], Lopez et al. [57], Mineshita et al. [58], Nabi & Wolfers [59], Nobusako et al. [60], Ribner et al. [61], Santaliestra-Pasías et al. [62], Shen et al. [63], Tay et al. [64], Zhu et al. [65]), according to the Joanna Briggs Institute (JBI) Critical Appraisal Checklist for Analytical Cross-Sectional Studies [37]. 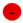 = no/unclear, 0 point; 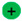 = yes, 1 point. Q1: Were the criteria for inclusion in the sample clearly defined? Q2: Were the study subjects and the setting described in detail? Q3: Was the exposure measured in a valid and reliable way? Q4: Were objective, standard criteria used for measurement of the condition? Q5: Were confounding factors identified? Q6: Were strategies to deal with confounding factors stated? Q7: Were the outcomes measured in a valid and reliable way? Q8: Was appropriate statistical analysis used?

Summary tables of quality assessments are included in this review in Figure 2 (RCTs), Figure 3 (cohort studies), and Figure 4 (cross-sectional studies). The risk of bias was assessed by using the Joanna Briggs Institute (JBI) Critical Appraisal Tool with a specific checklist for each study design (RCTs and cohort and cross-sectional studies). The overall quality of the included studies was “high” (n = 3), “moderate” (n = 33), and “low” (n = 4). The RCTs included in this review (n = 3) were rated as of moderate quality [66,67,68]; among cohort studies (n = 9), two were of high quality [70,72], whereas the remaining seven were of moderate quality; the cross-sectional studies (n = 28) were mainly rated as moderate (n = 23), followed by low (n = 4) and high (n = 1) quality.

The RCTs were rated as moderate-quality studies mainly due to performance biases (blinding procedures and exposure to other validity-threatening factors) and detection bias (blinding outcome assessors). Selection biases (allocation concealment, true randomization) further reduced the percentage of the quality score in two studies (Figure 2) [66,67]. Similarly, among the cohort studies (Figure 3), mainly rated as moderate-quality studies, several biases were reported, except for two studies [70,72], at risk of detection and attrition biases (validity of outcome measurements or incomplete outcomes due to follow-up withdrawals), though rated with a high quality score. Only 1 out of 28 cross-sectional studies (Figure 4) was rated as high-quality, though at risk of detection bias [60]; selection and recall biases (recalling past exposure, low accuracy) reduced the quality score of studies rated as “moderate”; thus, since multiple biases were detected, studies were rated with a low-quality ranking [39,44,50,64].

## 4. Discussion

The aim of this systematic review was to synthesize the evidence on the impact of digital device exposure on children’s physical and mental health. Moreover, some studies also investigated the effects of the lockdown period and e-learning methodologies on screen time and health indicators in children. The impact of digital device exposure was investigated considering five identified outcomes, namely physical activity and body composition, motor skills and posture, sleep habits, behavioral aspects, and COVID-19 and vision assessment. The overall results showed that exposure to digital devices affects various domains of children’s health. In this scenario, a better understanding of the relationships between clinical and emotional outcomes would increase our knowledge of protective or vulnerability factors associated with the development of negative emotions in these subjects interfaced with digital devices [80].

### 4.1. Generational Changes in Media Exposure

Among the included studies, the number of those evaluating behavioral and psychosocial outcomes exceeded other health parameters, demonstrating the importance of investigating the area of cognitive and mental domains of health at every life stage. However, there has been an increase in the number of publications during the past ten years, with a peak in 2021, mainly due to the COVID-19 pandemic. Among recent studies, Bohnert & Gracia [41] conducted a retrospective study to compare two cohorts with a large sample size, demonstrating a generational change in children’s exposure to digital devices: the 1998 birth cohort had higher levels of TV watching compared with the 2008 birth cohort, which, in turn, demonstrated a higher exposure to digital devices and social media. However, children are currently exposed to both TV and other digital devices (i.e., smartphones and tablets), with an anticipated first use (1–2 years old) compared to the past [44]. Trajectories (prospective evaluation) of media exposure were also evaluated by McArthur et al. [74], reporting that digital media habits are developed and maintained across early childhood. This is troubling considering the recent release of the WHO’s Guidelines on physical activity, sedentary behavior, and sleep for children under 5 years of age [81], in which screen time is not recommended (for 1 year-old children) or limited to no more than 1 h/d (for 2 year-old children) for healthy growth. As recently reported by Stiglic & Viner [16], public health guidelines for screen use are based on limited scientific evidence, and none of the studies used objective measurements (e.g., screen use measured with wearable cameras). However, the growing concerns of experts bring health institutions to aware parents and children on controlling digital media habits. In this regard, studies evaluating the proportion of children meeting guidelines about PA, sedentary behavior, and sleep reported poor compliance from children and awareness of parents involved [20,46]. Similar trends were comparable across countries, where a daily exposure higher than 2 h of screen time in more than 15,000 children was observed [61,62]. Therefore, due to the multicentric design of the studies and countries investigated and included in this review, the findings are generalizable across children of various communities and cultures.

### 4.2. Gender Differences and Socio-Demographic Influences on Media Habits

Gender differences did not emerge for digital device use, even if the impact of digital exposure elicited gender-related responses (i.e., mobile ownership affected more girls, whilst emotional regulation was reduced in boys engaged in digital sessions) [41]. Moreover, the quality of digital media engagement favored action-like mechanic gaming for males, who usually spend more time playing video games than females [43]. Of note, familiar context (i.e., socioeconomic status [SES], level of education, employment, etc.) influenced digital device exposure. Such variables were investigated as covariates in almost all of the included studies. Screen use increased with lower levels of parent education and SES [61], but, interestingly, parents’ screen habits were positively associated with a pattern of digital device use of their child [51,54] (TV-viewing time of parents was associated with TV-viewing time of children) [55]. Early childhood is particularly affected since media co-exposure and co-participation reduce with time [51]. This further highlights the need for guidelines promotion, primarily among parents, to increase awareness regarding screen exposure of children and change, where not appropriate, their digital media habits.

### 4.3. Screen Time, Timing, and Frequency of Media Use

Digital device exposure in 2-to-12-year-old children was found to be increased across time and countries [44,51,62]. In particular, digital media exposure showed an increasing trend as children aged [51] and due to the growing e-learning environment [44,51]. However, studies reported that the exposure rate was greater during the weekend and for entertainment purposes as compared to typical weekdays and educational apps [41,44,51,61,64]. Data were also available from other studies in which preschool children were usually exposed to digital devices during the weekend [82,83]. The specific content of digital media was not investigated among studies, representing a limiting factor of their approach and worth of future investigation [20,43,47,49,52,59,60,74,75,76]. The only study investigating digital media content and self-regulation skills in children reported significant differences between children preferring violent/horror content (poor self-regulation skills) as compared to educational content [42].

Children also show multitasking behavior by simultaneously using two or more digital devices, a tendency that increases with age [43]. Therefore, multitasking favored an increase in screen time per day, a reduction in non-digital time, PA engagement, sleep quality, behavioral regulation [56], and an increase in adiposity parameters (i.e., BMI) [57].

### 4.4. Physical Activity, Motor Skills, and Sleep Habits

PA engagement was lower among children exposed to a higher level of digital devices [51]. Furthermore, outdoor activities and sports club engagement were reduced in children exceeding their screen time exposure [62] or with direct access to more devices in personal spaces (i.e., bedroom) [49,62], additionally associated with higher values of BMI. Conversely, PA increased when digital devices were removed in children’s homes or replaced with active input games [68].

The presence of digital devices in children’s personal spaces increased the screen time as compared to children who had no access [45,49]. The bedroom is considered a personal space, and sleep quality is investigated considering the children’s exposure to digital devices in the bedroom before bedtime. Bright light emissions from screen exposure were previously reported to have a disturbing effect on sleep quality [84]. Indeed, the screen presence and exposure before bedtime affected sleep efficiency [45], sleep quality [49], and sleep duration with later bedtimes [49,52]. The risk of sleep disorders was also higher in children exposed to TV watching over 1 h/d but not in children who usually read a book before bedtime [65]. As for screen time recommendations, children seldom met the sleep guidelines, although it is strongly related to a healthy quality of life [77].

Digital media exposure did not differ regarding the types of devices, but a comparison between passive exposure and active interaction can be explored. Passive screen time is considered a non-interaction time in which screens are used (TV watching). On the other hand, the interaction with electronic devices determines an active screen time. According to studies, passive screen time induced a greater reduction in motor interaction and postural change when compared with digital and non-digital activities (playing sessions). Passive digital media exposure also reduced verbal information processing [76]. Conversely, both digital and non-digital activities promoted greater postural variations [54], even though digital conditions inhibited the so-called private speech (self-talk) as compared to non-digital or toy-playing conditions, inducing children’s isolation (“digital bubble effect”) and implications for executive functioning [40]. Therefore, interactive digital devices could be preferable to passive digital media exposure, though limited and proposed in parallel with toy play or non-digital activity sessions. However, active interaction (higher levels of app use) was associated with behavioral and psychological difficulties, similar to the poor regulatory behavior caused by TV watching [75]. Moreover, when comparing writing performances, digital keyboarding significantly differed from handwriting and stylus writing, but handwriting training on paper was comparable with handwriting with stylus in terms of letter acquisition and visual–spatial skill improvement [66,67]. It should also be stated that writing performances are partly involved in the overall cognitive development of children, for which studies reported significant and negative effects due to screen exposure. Indeed, sensory processing [48], manual dexterity [60], and cognitive development are reduced in children exposed to digital media and further reduces across time [73].

### 4.5. Behavior and Vision Assessment

Studies investigating behavioral changes and related parameters in children exposed to digital devices were the largest group of outcomes debated in this review. Both passive and active exposure to digital media provoked behavioral changes in children, such as a reduction in self-regulation [73], socioemotional functioning [73], and social adjustment [53], as well as poor academic performance [58] and psychological difficulties [58]. The negative impact of video games, such as frustration feelings for not winning, was also reported [50,85]. By contrast, Nabi & Wolfers [59] reported no associations between screen exposure and emotional intelligence, supporting the hypothesis that digital device exposure could not be related to the emotional development of children. Nonetheless, findings might also be limited by the study design, which is mainly cross-sectional and combined with a biased “parental perspective”.

Among the included studies, it was also possible to evaluate the impact of the COVID-19 pandemic since digital device exposure increased worldwide due to lockdown periods. In the time frame of prolonged lockdown, the screen time of children increased similarly across countries at an average of 1 h/d [61] before and after bedtime, thus affecting nocturnal sleep quality, especially among the girl sample [39]. As a consequence, scarce sleep quality can exert higher levels of depression, anxiety, and stress, naturally associated with extended periods of home confinement [86].

The main investigated outcome in the studies on the COVID-19 pandemic was vision risk. Indeed, various vision-related parameters (i.e., myopia progression, depth perception, visual acuity, etc.) were affected by the prolonged time of screen use and online learning [30,69,70,71]. Ocular pathologies were reported in association with the long-term exposure to blue light derived from digital devices. Moreover, short-term effects of dry eye sensation [58] and eye strain were detected due to screen light brightness [87].

### 4.6. Limitations

The quality score of the included studies was mainly rated as “moderate”, limiting the results of this systematic review. Moreover, it was not possible to conduct a meta-analysis with the included studies because fewer RCTs were available. Therefore, it is required to further explore the current issues with more experimental study designs. Indeed, a cross-sectional design was adopted in most studies with a prevalent use of surveys rather than an objective evaluation of outcomes. The limitations of the questionnaires for establishing digital device use were acknowledged by the authors. However, survey validations (e.g., SMALLQ^®^) [84] improve the reliability of such methodology, notwithstanding that an objective evaluation of media habits should be preferred for future studies. In this regard, various biases reduced the overall quality of studies, including recall biases and social desirability [48,51,63,65,73,77], especially in those evaluating “parental perspective”. Moreover, parent-reported information was partly supported by the association with sample socio-demographics that, when explored, provided a better understanding of family approaches to digital media habits. The age range of the children involved (2–12 years old), which partly forced the parental involvement (e.g., preschool children), should be accounted for. However, the large sample sizes of the multicentric studies support the evidence related to various aspects of children’s health.

## 5. Conclusions

This systematic review highlights a strong health impact of long-term exposure to digital devices and meaningful effects on 2-to-12-year-old children, such as PA reduction, sedentariness increase, and sleep disturbances. Behavioral changes in children overexposed to digital media were reported, such as socioemotional dysregulation and low levels of academic performance and cognitive development achievement. Following the lockdown periods, ocular problems were reported in children exposed to digital media due to online learning. Although the COVID-19 pandemic could be identified as a unique phenomenon, digital device overexposure remains stable worldwide with a growing trend.

## 6. Practical Recommendations

Considering the negative effects of prolonged exposure to digital devices on children’s health, a call-to-action should be proposed worldwide, especially among parents, to promote compliance toward screen time guidelines and increase awareness of screen exposure risks. Since WHO’s Guidelines are already available, strategies to promote correct digital device exposure among children should be implemented, and parents should be involved so they are aware of their children’s digital media exposure, considering risks for psychosocial, physical, and mental health and overall quality of life. For instance, strategies to promote healthy digital habits should include implementing screen-free times and/or non-digital activities. Schools could also be involved by supporting either digital literacy programs or promoting non-digital behaviors, while public health campaigns can emphasize the dangers of excessive screen time. Moreover, scientific research on effective interventions focused on screen habits will help create a healthier digital environment for children around the world.

## Data Availability

Data are available on request from the corresponding author.

## Supplementary Materials

The following supporting information can be downloaded at https://www.mdpi.com/article/10.3390/jfmk9040236/s1, Table S1: Data extraction synthesis of the included studies.
