# Supplementary material for: The Impact of Digital Devices on Children’s Health: A Systematic Literature Review
Source: J Funct Morphol Kinesiol. 2024 Nov 14;9(4):236. doi: 10.3390/jfmk9040236 (PMC11587142; doi:10.3390/jfmk9040236)
Supplement: Supplementary file 1 [file jfmk-09-00236-s001.zip › jfmk-3283626-supplementary.pdf]

Table S1. Data extraction synthesis of the included studies.

Reference	Country	Study design	Sample characteristics	Aim	Intervention/setting	Main findings	Quality score
Abid et al., (2021) [39]	Tunisia	Cross-sectional	5-12 years old (mean age 8.66 ± 3.3 years); N = 100, n = 52 M, n = 48 F	To investigate the effect of COVID-19 home confinement on sleep quality, digital media use and the level of PA.	Online survey based on before-and-after confinement question format: sleep quality was assessed with the Pittsburgh Sleep Quality Index (PSQI); frequency, time, and timing (during the day and 2 hours before bedtime) of digital media use were collected; level of PA was obtained with the Ricci and Gagnon sedentary behavior questionnaire.	Sleep quality was deteriorated during home confinement; PA levels decreased, and ST increased during the confinement. The effect of COVID-19 confinement was more evident in girls *vs* boys.	Low
Bochicchio et al., (2022) [40]	Italy	Cross-sectional	5-years preschool children (mean age 5.14 ± 0.25 years); N = 29 (white, Italian native); 52% F	To explore the production of PS during a material and digital version of the same identified task (ToL task).	ToL task completed in both material and digital versions Speech was encoded and recorded by researchers during the task.	PS decreased during the digital version of the ToL task as compared to the material version of the same game.	Moderate

Bohnert & Gracia, (2020) [41]	Ireland	Cross-sectional (retrospective cohort) study	2 cohorts of 9-year-old children (birth cohorts of 1998 and 2008); N = 13,397, 49% M, 51% F	To examine the patterns and effects of digital use on child socioemotional well-being across two cohorts of children.	Socioemotional well-being was measured with SDQ; screen time was calculated as time spent in TV watching and digital devices use on weekday; quality of digital engagement included gaming, leisure, education, social interaction, and personal development; technology family context was also investigated (i.e., parent supervision, mobile ownership).	TV screen time decreased between cohorts, while digital screen time increased (especially media/leisure activities); high levels of TV/digital screen time were associated with declines in socioemotional well-being; gender and SES elicit differences in media usage, but they did not moderate the effect of digital use on children's socioemotional well-being across cohorts.	Moderate
Canaslan-Akyar & Sungur, (2022) [42]	Turkey	Cross-sectional, observational study (survey research)	4-6 years preschool children (mean age 33.62 years); N = 911 (mothers); 49.2% M, 50.8% F	To examine self-regulation skill of 4-6-year-old children regarding digital media usage.	Survey research based on the self-regulation skills of 4-6-Year-Old Children (Mother Form) including 20 items assessing working memory, attention, inhibitory control-emotion, and inhibitory control-behaviour; screen time exposure was collected as daily amount in hours.	As the time spent with digital media tools increases, children’s total self-regulation scores were likely to decrease. Children who used a smartphone have lower levels of self-regulation; smartphone use did negatively impact the self-regulation skill of 4-6-year-old children. The children who preferred violent and horror content had lower levels of self-regulation than children who	Moderate

						did not have these preferences (favouring educational content).	
Cardoso-Leite et al., (2021) [43]	Switzerland	Cross-sectional	8-12 years children (mean age 10.38 ± 1.16 years); N = 118, n = 61 M, n = 57 F	To investigate three different ways of measuring technology use - total hours of media consumed, hours of video game play, and number of media used concurrently (media multitasking) - and their relationship with attention, behaviour, mental health, sleep, grades.	Children demographics were self-reported; parents and/or teachers completed questionnaires on (i) children's media usage, (ii) attentional and behavioural control abilities (with additional cognitive task, SDQ), (iii) psychological distress, psychosocial functioning, and sleep, and (iv) academic achievement and motivation.	Digital media usage and numbers of additional media devices increase as children age (one additional hour per year of age). Gender differences detected only for types of media consumed (boys prefer action-like mechanics video game). Media multitasking negatively affected sleep, behaviour, attention, grades, socioemotional functioning with higher levels of psychological distress.	Moderate
Chang et al., (2018) [44]	Korea	Cross-sectional	2-5 years children (mean age 3.85 ± 0.7 years); N = 390; 52.3% M	To investigate the exposure of smart devices among 2–5 years old children.	Via survey, parents reported demographics of their child; type of devices and children's frequency of media use were collected. It was also	TV and smartphones are the most popular digital devices used by toddlers, who begin using smart devices very early (1-2 years old). Screen media use was prevalent in weekend days rather than weekdays. TV watching has	Low

					asked the child's age at the first use of media.	a decreasing trend as smart devices use, and availability are growing.	
Chaput et al., (2014) [45]	Canada	Cross-sectional	9-11 years children (mean age 10.0 ± 0.4 years); N = 502; 41% M, 59% F	To examine whether the number and type of electronic screens available in children's bedrooms matter in their relationship to adiposity, physical activity, and sleep.	Parent-reported screens presence in children's bedrooms; children self-reported their screen time. Body composition was assessed with bioimpedance, and accelerometer was used to assess the amount of physical activity, sedentary time, sleep duration and efficiency over 7 days.	The higher number of screens in children's bedrooms is associated with higher adiposity (TV especially among the other types of screens), more total screen time and lower sleep efficiency.	Moderate
Chaput et al., (2017) [20]	Canada	Cross-sectional	3-4 years (mean age 3.5 years); N = 803; n = 400 M, n = 403 F	To examine the proportions of preschool-aged (3 to 4 years) children who met movement guidelines and recommendations, and the associations with adiposity indicators.	Screen time and sleep duration were parents/guardians-reported; physical activity was assessed by accelerometer, and BMI status and z-scores were reported as adiposity indicators.	Very few preschool-aged children in Canada (about 13%) met all three recommendations contained within the *24-Hour Movement Guidelines*[a]. None of the combinations of recommendations were associated with adiposity.	Moderate
Chia et al., (2020) [46]	Singapore	Cross-sectional	2-4 years (gender, mean age, and sample size not reported)	To determine the proportion of preschool children under 5 years old meeting the 24-h	Parent-reported online questionnaires: Surveillance of screen Media hAbits	9.6% of preschool children met all three integrated 24-h guidelines on physical activity, sedentary behaviour, and sleep and 12.6% met	Moderate

				World Health Organization guidelines[b] on physical activity, sedentary behaviour, and sleep. To examine the association between the health-related quality of life and meeting these guidelines.	in earLy chiLdhood Questionnaire (SMALLQ®); the Pediatric Quality of Life 4.0 Inventory (PedsQL™ 4.0)	none of the guidelines. The health-related quality of life of preschool children increased with the number of WHO guidelines accomplished.	
Cox et al., (2012) [47]	Australia	Cross-sectional	2-6 years (mean age 4.5 ± 0.84 years); N = 135; n = 54 (40%) M, n = 81 (60%) F	To explore the relationships between preschool children's TV viewing habits (i.e., time spent viewing, content watched, and foods eaten while viewing), daily food intake, physical activity levels, and body mass index (BMI).	Parent-reported 3-day TV diary (viewing time, content, food consumed while watching TV), height, weight, and physical activity behaviour; BMI and BMI z-score were calculated starting from parent-reported height and weight of children.	Preschool children's TV watching was associated with higher BMI, maybe due to displacement of physical activity and/or increased energy intake during viewing.	Moderate
Dadson et al., (2020) [48]	Australia	Cross-sectional, exploratory study	4-7 years (mean age 6.2 ± 1.03 years); N = 25; n = 9 M, n = 16 (64%) F	To explore the association between children's screen-time, fine motor, inhand manipulation (IHM), visual-motor integration (VMI), sensory processing	Parent/Caregiver-reported survey on daily screen-time; Bruininks–Oseretsky Test of Motor Proficiency—Second Edition, Test of In-Hand Manipulation—	Statistically significant negative correlations were found between children's screen-time, VMI, fine motor skills, IHM, SP and enjoyment of play. Screen-time also had a significant negative association with children's SP skills.	Moderate

				(SP) and parent-reported play skills.	Revised, Beery Buktenica Developmental Test of Visual-Motor Integration Sixth Edition, Sensory Processing Measure—Home Form and Pretend Play Enjoyment Developmental Checklist (PPEDC).		
Dube et al., (2017) [49]	Canada	Cross-sectional	10-11 years; N = 2,334; n = 1,071 M, n = 1,235 F	To assess the impact that the use of electronic entertainment and communication devices (EECDs) before bedtime has on sleep and weight status.	Parent-reported (survey) sleep habits, presence, and usage of devices in the hour before bedtime; weight status and BMI evaluation.	Sleep duration, sleep quality, sleep efficiency and weight status are better among children who do not have EECDs in the bedroom and frequently read a book during the hour before sleep versus those who use EECDs during this hour.	Moderate
Gonzalez-Valero et al., (2019) [50]	Spain	Cross-sectional	11-12 years (mean age 11.41 ± 0.5 years); N = 577; n = 328 (56.8%) M, n = 249 (43.2%) F	To establish the relationships between the problematic use of video games with physical-healthy and psychosocial variables.	Ad-hoc questionnaire to register gender, age, daily physical activity, sleeping hours and use of digital screens; Adherence to the Mediterranean diet (KIDMED); Questionnaire of Experiences Related to Videogames; BMI	The students with higher values of digital screen use show severe problems with video games, poor self-concept, sleep problems, and higher values of BMI.	Low

					calculated with bioimpedance.		
Hasanen et al., (2021) [51]	Finland	Cross-sectional	2-6 years (mean age 4.71 ± 1.37 years); N = 2,512; 52% M, 48% F	To examine the digital media use (DMU) and physical play in early childhood, parental co-participation, and sociodemographic correlates.	Parent-reported online questionnaires: Surveillance of screen Media hAbits in earLy chiLdhood Questionnaire (SMALLQ®).	The DMU increased with age, and the hours spent in DMU are higher as compared to outdoor physical play. Parental co-participation in both activities decreased as children became older, but it correlates positively with DMU and physical activity.	Moderate
Hiltunen et al., (2021) [52]	Finland	Cross-sectional	3-6 years (mean age 4.7 ± 0.89 years); N = 736, n = 381 (52%) M, n = 355 (48%) F	To examine the associations between screen time, the time spent on different screen devices and sleep.	Parent-reported 7-day diary about screen time, types and number of devices; sleep habits as bedtime/wake-up time, sleep duration and consistency (sleep regularity with Children's Sleep Habits Questionnaire, CSHQ).	The higher amount of total screen time was associated with later bedtimes and shorter sleep duration, without gender differences.	Moderate
Hosokawa & Katsura, (2018) [53]	Japan	Cross-sectional	5-6 years; N = 1,642, n = 841 (51.2%) M, n = 801 (48.8%) F	To clarify the association between the use of mobile devices and emotional/behavioural problems.	Parent-reported the Strength and Difficulties Questionnaire (SDQ) about children's prosocial and difficult behaviours;	The excessive use of mobile devices, including smartphones and tablets, might interfere with children's development in relation to social adjustment.	Moderate

					mobile use average time on a typical day.		
Howie et al., (2017) [54]	Australia	Cross-sectional laboratory trial	3-5 years (mean age 4.1 ± 0.8 years); N = 10, n = 4 M, n = 6 F	To examine body postures and physical activity/sedentariness during playing with tablet, TV watching, and non-screen toys play.	Vicon system for motion analysis in three conditions: play with tablet, TV watching, and toy play condition. Evaluation of arm, trunk, and head variations during the three conditions.	The use of touch screen devices increased musculoskeletal disorder risk, sedentary behaviour and reduced physical activity, as compared to non-screen toy play.	Moderate
Hu et al., (2021) [69]	China	Cohort study	7-8 years (mean age 7.76 ± 0.32 years); N = 2,679, n = 1,422 (53.1%) M, n = 1,257 (46.9%) F	To investigate changes in myopia development during the outbreak of COVID-19.	Changes in cycloplegic spherical equivalent refraction (SER), axial length (AL) elongation, and myopia incidence from grade 2 to grade 3 children (from 2018 to 2020).	Myopia increased in children exposed to COVID-19 period along with the proportion without myopia but with a risk of developing the condition.	Moderate
Jago et al., (2012) [55]	Portugal	Cross-sectional	3-10 years; N = 2,965, 51.2% F	To examine if parental screen-viewing time and electronic media, environment factors were associated with children's screen-viewing habits.	Parent-reported screen-viewing time of both father and mother, and of their child on typical weekday and weekend day; presence and position of media equipment at home.	Parental TV-viewing time was strongly associated with young children's TV-viewing time (no gender differences detected).	Moderate
Kiefer et al., (2015) [66]	Germany	Randomized control trial	4-6 years (mean age 5 ±	To investigate the influence of	Training program of 16 sessions in which	Results favoured the handwriting training over the	Moderate

			4 months); N = 23, n = 11 M, n = 12 F	handwriting and typewriting training on letter recognition, reading and writing performance in matched groups of preschool children.	children were trained in handwriting or typewriting.	typing training in word writing, and, as a tendency, in word reading tasks.	
Kostyrka-Allchorne et al., (2020) [56]	England	Cross-sectional	3-11 years; N = 520, n = 261 (50.2%) M, n = 259 (49.8%) F	To examine the associations between screen use, non-digital recreation, and childhood psychopathology symptoms.	Parent-reported Strength and Difficulties Questionnaire (SDQ), information about the non-digital recreation and digital media use/media multitasking.	Children are either less likely to participate in non-digital recreation and they are more likely to use screen media or multitask with media. Psychological and behavioural difficulties occurred with reduced sport participation, less reading and games, and increased media use.	Moderate
Lopez et al., (2019) [57]	USA	Cross-sectional	9-11 years (mean age 9.93 ± 0.58 years); N = 179, n = 91 (51%) M, n = 88 (49%) F	To examine the association between children's media multitasking tendencies and adiposity.	Adaptation of Media Multitasking Inventory (reporting information about multitasking in print and digital media use); BMI and BMI z-score were calculated on digital scale and used to assess adiposity.	There was a statistically significant positive relationship between the frequency of media multitasking behaviours and BMI (adiposity).	Moderate
Ma et al., (2021) [70]	China	Cohort study	8-10 years (mean age 8.9 ± 0.69 years); N = 208, n = 109 (52.4%) M	To determine myopia progression and the related factors associated during the COVID-19.	Ocular examination (baseline, before and during COVID-19); questionnaire on myopia risk factors.	Children were at higher risk of myopia progression during COVID-19, which was associated with the axial length, the long-time online	High

						learning, and digital screen reading.	
Ma et al., (2021) [71]	China	Cohort study	7-12 years (mean age 9.9 ± 1.7 years); N = 201, 48% M	To investigate the effect of home quarantine during the COVID-19 pandemic on myopia progression.	Clinic examination (baseline, before, and after 4 months of COVID-19 quarantine) to assess myopia (visual acuity, ocular pressure, etc.); parent-reported questionnaire on the average time spent in digital screen devices.	COVID-19 home quarantine generated changes in behaviour and myopic progression, associated with digital screen use (tablets and mobiles, lesser with TV and projectors).	Moderate
Madigan et al., (2019) [72]	Canada	Cohort study	2-5 years; N = 3,089, n = 1,622 (47.9%) M, n = 1,467 (43.3%) F	To assess the directional association between screen time and child development.	Ages and Stages Questionnaire, Third Edition (ASQ-3) to assess developmental progress in 5 domains (communication, gross motor, fine motor, problem solving, and personal-social); screen time use in hours/week.	There was a directional association between screen time and poor performance on development screening tests, especially among 2-3-year-old children.	High
Martzog & Suggate, (2022) [73]	Germany	Cohort study	35-82 months (2.9-6.8 years) preschool children (mean age 4 years and	To investigate directional links between screen media usage and the development of fine	Parent-reported media usage (on a typical day and on the weekend), Movement-	Screen media usage (mobiles, computer, tablet, etc. but not television) is negatively associated with	Moderate

			10 months ± 11 months); N = 141, 50% M	motor skills, accounting for inter-individual differences and key covariates.	Assessment-Battery for Children (Movement ABC) to assess fine motor skills.	FMS development across time.	
Mayer et al., (2020) [67]	Germany	Randomized control trial	4-6 years (mean age 5 years and 10 months ± 5.2 months); N = 147 (n = 49, pencil group; n = 50, stylus group; n = 48, keyboard group), n = 75 F	To test the influence of various writing tools (handwriting on a paper sheet, handwriting on a tablet with stylus, and digital keyboard) on the acquisition of literacy skills.	Training program of 7 weeks in which children were trained to handwriting with pencil on paper, with stylus on a tablet, and typing letters on a virtual keyboard. Tests on writing performances were provided before, after, and 4-5 weeks after training.	Handwriting with pencil fosters acquisition of letter knowledge and improves visuo-spatial skills compared with keyboarding. The stylus group did not differ significantly neither from the keyboard nor from the pencil group.	Moderate
McArthur et al., (2020) [74]	Canada	Cohort study	2-5 years; N = 1,949, n = 996 (51.1%) M, n = 914 (46.9%) F	To identify trajectories and patterns of screen use, socio-demographic factors, child behaviour, and learning outcomes associated between the ages of 24 and 60 months.	Mothers reported the screen use of their child on weekdays/end at 24, 36, and 60 months of age; child behaviour was assessed by the Behavior Assessment System for Children (BASC-II); Ages and Stages Questionnaire, Third Edition (ASQ-3) was used to assess developmental progress; covariates	The 9.5% of sample had a high-persistent screen use, the 90.5% of sample had a low to moderate screen use trajectory; screen use patterns (about 4 hours/day) were maintained across the early childhood, with a peak at 36 months. Poor performance in children's developmental achievement and low level of adaptive behaviour were associated to higher levels of screen use.	Moderate

					(gender, economic income, maternal characteristics, etc.) were investigated.		
McNeill et al., (2019) [75]	Australia	Cohort study	from 3-5 years to 4-6 years (mean age 4.2 ± 0.6 years); N = 185, n = 112 M, n = 73 F	To investigate associations of traditional (program viewing) and contemporary (electronic applications, or apps) electronic media use with pre-schoolers' executive function and psychosocial development 12 months later.	Parent-reported number and types of electronic media in the house and total time spent in media use on weekdays/end; executive functions were assessed with the Early Years Toolbox (EYT); psychosocial health was measured with Strengths and Difficulties Questionnaire (SDQ); covariates were investigated via parent-report survey.	Higher levels of app use and program viewing (TV or any device) were associated with behaviour problems and psychological difficulties, and lower inhibition at follow-up, respectively.	Moderate
Mineshita et al., (2021) [58]	Japan	Cross-sectional	6-12 years; N = 7,419 (gender demographics and mean age differed among the investigate variables)	To examine the effect of screen time duration and timing effects on obesity, physical activity, dry eyes, and learning ability.	Teacher and parents were asked to complete questionnaires about children learning ability and demographics, respectively;	Screen time duration and timing were both associated with obesity, and academic performance; duration of screen time was additionally associated with physical activity and timing with dry eyes. Screen time	Moderate

					children reported their habits about (i) physical activity ,with the WHO Health Behavior School-Aged Children (HBSC), (ii) screen time timing and (iii) duration; BMI percentile was used to assess obesity; Dry Eye-Related Quality-of-Life Score (DEQS) questionnaire to assess dry eye sensation.	immediately before bedtime increases the risk of dry eyes, and longer screen time duration can contribute to reduction in academic performance.	
Nabi & Wolfers, (2022) [59]	n/r	Cross-sectional, exploratory study	5-12 years (mean age 8.2 ± 2.54 years); N = 400 (parents), 55% M, 45% F	To assess how the media diet of children and the media use of their parents relates to child emotional intelligence (EI)[c] and if any specific patterns exist.	Parents assessment of self-EI and EI of their child/ren, empathy, emotional regulation, self-reported and child media use and non-media activities, media co-use, emotional mediation; control variables were also investigated (gender, child age).	No screen or digital media use significantly related to child EI, empathy, or emotional regulation, though reading did associate with higher EI. Concerns about children's digital or screen media use are perhaps overblown in terms of impeding emotional skill development.	Moderate

Nobusako et al., (2020) [60]	Japan	Cross-sectional	6-12 years (mean age 9.2 ± 1.9 years); N = 100, n = 40 M	To investigate the association between media viewing time and preference level, perceptual bias, and manual dexterity.	Media viewing time and preferred level were assessed by both parents and children via questionnaires; children were additionally asked to complete visual-tactile temporal order judgment (TOJ) task, and manual dexterity test with the Movement Assessment Battery for Children - 2nd edition (M-ABC-2).	Significant associations were found between age and media viewing time, between media viewing time and media preference level, and between visual bias and manual dexterity. Increased visual bias was a significant predictor of reduced manual dexterity.	High
Ribner et al., (2021) [61]	Australia, China, Italy, Sweden, United Kingdom, USA	Cross-sectional	3-7 years (mean age 5.77 ± 1.10 years); N = 2,516, 50.6% M, 47.9% F (1.5% no response)	To evaluate changes in electronic screen-based media use during the COVID-19 pandemic.	Parent-reported amount of media use, time and type of device used and media content; COVID-19 exposure and social disruption (i.e., job loss, financial strain) were also investigated; family SES and other variables (child gender, age, etc.) were controlled as covariates.	Across Countries, parents reported an average increase of nearly an hour of screen time per day for entertainment purposes (as compared to educational app). The lower SES, rather than the higher, increased screen use.	Moderate

Santaliestra-Pasías et al., (2014) [62]	Belgium, Cyprus, Estonia, Germany, Hungary, Italy, Spain, Sweden	Cross-sectional	2-10 years; N = 15,330; 51% M	To estimate the prevalence of physical activity and sedentary behaviours, and to evaluate the relationship between media availability in personal space and physical activity in relation to total screen time.	Parent-reported information about screen time (on weekend and weekday according to the AAP recommendations[d]), device availability (numbers of screen and type) in personal space, child engagement in sports club and/or outdoor physical activity.	Across Countries, a third of the children were engaged in total screen time > 2 h/d, especially the older children (6-10 years old) and during weekend days. Media availability in personal spaces (i.e., bedroom) favoured the excess in total screen time.	Moderate
Shen et al., (2021) [63]	United Kingdom	Cross-sectional	11-12 years (median age 12.06 years); N = 6,616 , n = 3,147 (47.57%) M, n = 3,469 (52.43%) F	To examine the associations between digital technology use (phone calls, internet use, video gaming) and measured BMI outcomes; mediation of insufficient sleep was also investigated.	Phone calls, internet use, and video gaming activities as well as sleep habits were reported by participants and collected; BMI z-scores were obtained with bioimpedance analysis; age, gender, ethnicity, parental education, occupation, and dietary factors were controlled as covariates.	There was a significant association between digital technology use and BMI in adolescents; BMI z scores were more strongly related to high weekday digital technology use; insufficient sleep mediated partially the BMI z scores.	Moderate
Straker et al., (2013) [68]	Australia	Crossover randomized control trial	10-12 years (mean age 11.3 ± 0.8 years); N = 56, n = 29 F	To evaluate the impact of the removal of home access to traditional electronic	8-week intervention with electronic games removal or replacement with	The removal of electronic games in children's homes or replacing them with active electronic games resulted in	Moderate

				games or their replacement with active input electronic games on daily physical activity and sedentary behaviour.	traditional electronic games or "active" games.	increased PA and reductions in sedentary time during after-school time.	
Tay et al., (2021) [64]	Singapore	Cross-sectional	2-7 years; N = 3,413 (survey responses of which 2,016 were from parents of 2 to 4 children and 1,397 were from parents of 5 to 7 children)	To investigate parents' attitudes and concerns towards the use of technology and digital media.	Surveillance of digital Media hAbits in earLy chiLdhood Questionnaire (SMALLQ®).	Laptops, tablets, and mobile phones are the prevalent devices at home. Parents strongly influence the digital behaviour of their children. The greatest amount of screen time is about entertainment purposes (as compared to learning app).	Low
Veraksa et al., (2021) [76]	Russia	Cohort study	from 5-6 years to 6-7 years (mean age 5.72 ± 0.33); N = 122, 54.9% M, 45.08% F	To examine the relationship between phonological memory and passive (watching TV) and active screen time with Smart Screen Technologies (tablets, phone).	The Understanding of Similar Sounding Words was used to assess the phonological memory, Raven's matrices for the non-verbal fluid intelligence; passive and active screen time was obtained with survey to mothers; demographics of children and mothers were collected.	Passive screen time exposure (TV watching), rather than interactive smart screen technologies, negatively impact on child's ability to process verbal information. No-long term effects of both passive and active interacts were detected over a year.	Moderate

Zhang et al., (2020) [30]	China	Cohort study	mean age 10.02 years; N = 783 (n = 721 experimental group, n = 62 control group), n = 402 M, n = 381 F	To assess vision parameters (visual acuity, visual field, depth perception, and horary visual acuity), related differences and developmental changes in the E-learning environment.	Evaluation of visual acuity, visual field, depth perception, and horary visual acuity at 3 timepoints assessment during a 2-year follow up.	Poor vision was detected in growing children living in a E-learning environment. Duration and frequency of E-learning had no main influence on vision and depth perception, but it can exert long-term deterioration effect on vision with gradual progression.	Moderate
Zhu et al., (2020) [65]	China	Cross-sectional	3-6 years; N = 2,278, 50.7% M, 49.3% F	To examine the dose-response association between time spent on different electronic devices and children's sleep disorder.	Sleep disorders were determined with the Children's Sleep Habits Questionnaire (CSHQ) (parent-reported questionnaire); data on screen time and devices type (TV, laptop, tablet, phone) were also collected. Confounders variables were controlled (child age, gender, parent's smoking habits, education, etc.)	It was reported a J-shaped association between TV watching time and sleep disorder, with higher risk of sleep disorder over the threshold of 1 h per day and related increment for each additional hour spent in TV watching (+12%).	Moderate

[a]*Canadian Movement Guidelines*: at least 180 min of physical activity (of which at least 60 min is moderate-to-vigorous physical activity), engagement in no more than 1 h of screen time, and sleep duration between 10 and 13 hours.

[b]*The 24-WHO Guidelines*: having at least 180 min of physical activity, engaging in less than 60 min of screen media, having 10–13 h of good quality sleep.

[c]EI is a set of mental abilities that allows a person to recognize and effectively regulate emotional states, and to use emotions to plan, motivate, and achieve goals.

[d]The American Academy of Paediatrics (AAP) recommends media time exposure of $\leq$ 2 h/d for mitigating negative health effects in youth people.

BMI = body mass index, DMU = digital media use, EECDs = electronic entertainment and communication devices, EI = emotional intelligence, F = female, FMS = fine motor skills, M = male, mean age ± standard deviation, n/r = not reported, PA = physical activity, PS = private speech, namely self-talk children use during play, SES = socioeconomical status; SDQ = Strengths and Difficulties Questionnaire, ST = screen time, ToL = Tower of London, WHO = World Health Organization
